# Molecular mechanisms of synaptogenesis

**DOI:** 10.3389/fnsyn.2022.939793

**Published:** 2022-09-13

**Authors:** Cai Qi, Li-Da Luo, Irena Feng, Shaojie Ma

**Affiliations:** ^1^Department of Neuroscience, Yale School of Medicine, New Haven, CT, United States; ^2^Department of Cellular and Molecular Physiology, Program in Cellular Neuroscience, Neurodegeneration and Repair, Yale University School of Medicine, New Haven, CT, United States; ^3^Boston University School of Medicine, Boston, MA, United States

**Keywords:** scaffold protein, cell adhesion molecule (CAM), cytoskeleton, neural activity, evolution, disease

## Abstract

Synapses are the basic units for information processing and storage in the nervous system. It is only when the synaptic connection is established, that it becomes meaningful to discuss the structure and function of a circuit. In humans, our unparalleled cognitive abilities are correlated with an increase in the number of synapses. Additionally, genes involved in synaptogenesis are also frequently associated with neurological or psychiatric disorders, suggesting a relationship between synaptogenesis and brain physiology and pathology. Thus, understanding the molecular mechanisms of synaptogenesis is the key to the mystery of circuit assembly and neural computation. Furthermore, it would provide therapeutic insights for the treatment of neurological and psychiatric disorders. Multiple molecular events must be precisely coordinated to generate a synapse. To understand the molecular mechanisms underlying synaptogenesis, we need to know the molecular components of synapses, how these molecular components are held together, and how the molecular networks are refined in response to neural activity to generate new synapses. Thanks to the intensive investigations in this field, our understanding of the process of synaptogenesis has progressed significantly. Here, we will review the molecular mechanisms of synaptogenesis by going over the studies on the identification of molecular components in synapses and their functions in synaptogenesis, how cell adhesion molecules connect these synaptic molecules together, and how neural activity mobilizes these molecules to generate new synapses. Finally, we will summarize the human-specific regulatory mechanisms in synaptogenesis and results from human genetics studies on synaptogenesis and brain disorders.

## Introduction

The word “synapse,” coined by Charles Sherrington, is used to describe the place at which communication occurs between neurons. The existence of the synapse was consolidated by electron microscopy after years of debate (De Robertis and Franchi, 1956; Sotelo, 2020). The study of synapses started with histology and was later joined by biophysics, molecular biology, and biochemistry. It is now well-recognized that there are two kinds of synapses in the nervous system: electrical and chemical synapses. The electrical synapses allow an ensemble of cells to be activated simultaneously, as compared to chemical synapses where there is a delay in the transmission of signals. There has been accumulating evidence supporting the importance of electrical synapses in physiological processes (Pereda, 2014), but here we will primarily review the molecular mechanisms of the synaptogenesis of chemical synapses. Synapses are composed of three parts: the pre-synaptic terminal, synaptic cleft, and post-synaptic compartment. Synapses transform the electrical signals into chemical signals and then into electric signals again. They help living animals survive their environments, are the targets of predators, and are often affected under diseases. Consequently, understanding the molecular genetic basis of synaptogenesis may help us understand how genes instruct the wirings of the nervous system and direct the flow of information (Colon-Ramos, 2009), how the environment shapes behaviors, and how diseases arise, especially in humans.

Synapse formation involves the interactions between neurons. As a result, neuron differentiation, migration, axon guidance, and dendrite morphogenesis can all influence the outcomes of synaptogenesis (Batool et al., 2019). Nevertheless, even if all these processes are accomplished, forming synapses still requires precise regulations (Chia et al., 2013). To gain insights into the underlying mechanisms required to generate a synapse, one needs to first know the components that constitute a synapse and how they are assembled together. Both pre- and post-synaptic compartments contain electron-rich structures which are named the “cytomatrix” and “post-synaptic density,” respectively. These structures consist of mostly scaffold proteins that are connected intercellularly by cell adhesion molecules and are anchored by the cytoskeleton intracellularly. Accordingly, forming synapses usually requires a few prerequisites. First, synaptic molecules must be assembled at pre- and post-synaptic sites instead of randomly in the cytosol. Second, pre- and post-synaptic compartments where these synaptic molecules dwell must be aligned and connected accurately by cell adhesion molecules. Third, the cytoskeleton must be organized and coordinated so as to support the synaptic structure. In addition, synapses are dynamic and can be influenced by neural activities. Thus, by manipulating neural activities, we can examine the molecular events that impact the destinies of synapses. Last but not least, synaptogenesis, though largely conserved, has been constantly reshaped during evolution (Schmidt and Polleux, 2021), which endows humans with unprecedented cognitive capabilities and provides avenues to develop treatments and therapies for neurological disorders and diseases.

To better parse the process of synaptogenesis, we have organized this review into four parts: (I) the assembly of intracellular synaptic proteins, most of which are scaffold proteins (Jin and Garner, 2008; Sheng and Kim, 2011); (II) cell adhesion molecules that bridge these proteins intercellularly (Sudhof, 2021); (III) the cytoskeleton system that supports the structure of the synapse; and (IV) neural activity that drives the formation of new synapse (Zito and Svoboda, 2002; Pan and Monje, 2020). These aspects coordinated with each other, were renovated during evolution, and, when gone awry, are frequently associated with impaired brain formation or cognitive abilities. We will review each of these aspects using some well-studied examples and summarize their relationships to the development of diseases.

## Assembly of scaffold proteins and synaptogenesis

### Identification of synaptic components and their interactomes

To study the molecular mechanisms of synapse formation, the first step is to identify the molecules that constitute the synapses and examine their ways of assembly through protein interactions during synaptogenesis, because most of them are used to define synapses. Studies on synaptic transmission led to the observations that the pre-synaptic terminals contain ready-to-be-released vesicles docking on the “cytomatrix” and that post-synaptic compartment named post-synaptic density (PSD) contain “patches” that are electron-enriched (Phillips et al., 2001). Endeavors to purify synaptic proteins and examine their effects on synaptic transmission, led by Sudhof et al. (1993), discovered synaptic vesicle proteins such as synaptophysin, synapsin, synaptobrevin/VAMP 1/2, syntaxin, and SNAP-25 as well as their functions in synaptic transmission. Although genetic evidence from knockout mice suggests that most of them have no effects on the structure of synapses (Rosahl et al., 1995; Ahnert-Hilger et al., 1996; Fujiwara et al., 2006; Kwon and Chapman, 2011), they have laid the foundation for further probing the building blocks of synapses that enable the vesicles to dock. Advances in biochemistry, molecular biology, immunology, and other fields such as imaging (Fortin et al., 2014) have provided versatile approaches to dissect the building blocks of synaptic components and describe the protein meshwork for synaptic transmission, transforming the search into a more feasible and fruitful field of study (Sudhof, 1995; De Camilli and Takei, 1996).

**FIGURE 1:**
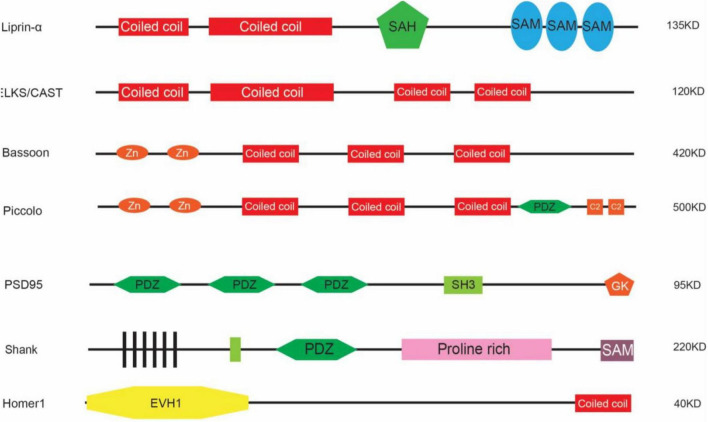
Schematic illustration of representative scaffold proteins (not scaled).

One approach is genetic screening. For example, using *Synaptobrevin*/VAMP fused with GFP (Hallam and Jin, 1998; Nonet, 1999), Zhen and Jin (1999) performed screenings on *C. elegans* regarding the localization of synaptic vesicles and discovered *Liprin-α* which was also named *syd-2* (short for synaptic defective). *Liprin*-α encodes a synaptic scaffold protein and was first identified through the yeast-two-hybridization as a binding protein for the leukocyte common antigen-related protein (LAR) transmembrane protein (Serra-Pages et al., 1995, 1998). There are four members of *LIPRIN-α* in humans with distinct expression patterns: *LIPRIN-α1* and *LIPRIN-α4* are widely expressed across the body, whereas *LIPRIN-α2* and *LIPRIN-α3* are enriched in the brain (Serra-Pages et al., 1998). LIPRIN-α contains multiple domains including two coiled-coil (CC) domains, a single alpha helix (SAH) domain, and three sterile-α-motif (SAM) domains (Figure 1). The N-terminal coiled-coil domains are sufficient to mediate the homo- and heterodimerization between liprins, and C-terminal regions interact with LARs (Serra-Pages et al., 1995, 1998). When co-expressed together with LARs in cell lines, Liprin-α was sufficient to promote the clustering of LARs (Serra-Pages et al., 1995, 1998), suggesting that through oligomerization, LIPRIN-αs provided a platform for the assembly of other synaptic proteins. They interact with multiple synaptic proteins that are essential for the development of both pre- and post-synaptic compartments (Spangler and Hoogenraad, 2007; Xie et al., 2021). At the pre-synaptic site, SYD-2/Liprin-α recruits the synaptic protein ELKS/CAST to promote the assembly of the active zone (Dai et al., 2006). ELKS is a protein around 120 KD and contains four coiled-coil domains indicating its role in associating with other protein components. CAST is also a member of this protein family that is structurally homologous but with a differential expression pattern as compared to ELKS in that CAST is highly expressed in the brain whereas ELKS is broadly expressed (Hida and Ohtsuka, 2010). ELKS interacts with Liprin-α through the coiled-coil domain and mediates the function of Liprin-α in recruiting synaptic vesicles to the active zone in worms (Dai et al., 2006). Indeed, ELKS/CAST can bind with RIM1 (Rab interacting molecules) and regulates synaptic transmission, consistent with its role in the generation of functional synapses (Takao-Rikitsu et al., 2004). ELKS also interacts directly with Bassoon and Piccolo through the coiled-coil domain (Takao-Rikitsu et al., 2004), suggesting that it plays a pivotal role in the assembly of active zone proteins. Piccolo and Bassoon were identified from a study by Craig Garner in 1996 in which a cDNA library from the rat brain was screened with antisera against the synaptic fraction and the positive clones were selected as candidates for synaptic proteins (Garner et al., 1988; Langnaese et al., 1996). They are structurally related proteins both containing zinc-finger regions and coiled-coil domains (Takao-Rikitsu et al., 2004). Through the same approach, a few other synaptic proteins were discovered in addition to Piccolo (Cases-Langhoff et al., 1996; Fenster et al., 2000) and Bassoon (Dieck et al., 1998), such as SAP90 (also known as PSD95) (Kistner et al., 1993), SAP97 (Muller et al., 1995), and SAP102 (Muller et al., 1996).

Another approach is homologous cloning of genes identified from other model organisms or tissues such as *C. elegans* or fruit flies. For example, the mammalian homolog of *unc-13, Munc-13*, was found in this way (Brose et al., 1995); identified and cloned by Brenner (1974) and Maruyama and Brenner (1991), *unc-13* causes abnormal neurotransmitter release but normal anatomical structure when mutated in worms (Richmond et al., 1999). In mammals, there are three isoforms of *Munc-13* that are enriched in the brain. However, neither *Munc-13-1* nor *Munc-13-2* has been found to be essential for synaptic morphogenesis in mice (Rosenmund et al., 2002). Using known synaptic proteins as baits to probe unknown candidates is also an effective strategy. For example, by fusing the cytoplasmic domain of SYNTAXIN A with glutathione-*s*-transferase (GST) and performing pull-down assays followed by amino acid sequencing, *Munc-18/STXBP1* was discovered (Hata et al., 1993b); through yeast two-hybrid screen using the C-terminal of NEUREXIN, *Cask* was cloned (Hata et al., 1996); and using the active mutant of Rab3C or its isolated domains, the active zone protein RIM (Rab3 Interacting Molecules) and its interaction partners such as RIM-BP, ELKS, and CAST were identified (Wang et al., 1997, 2000, 2002) [sub-cellular fractionation of mammals’ synaptic fraction also independently discovered CAST (Ohtsuka et al., 2002)]. Among these molecules, mice deficient in *Munc18-1* die immediately after birth but the differentiation of synapses appears normal (Verhage et al., 2000). Cultured neurons from Cask knockout mice developed a similar number of synapses when compared with wild-type counterparts, indicating that Cask is not essential for the formation of structurally sound synapses (Atasoy et al., 2007). Studies on the morphology from cultured neurons where RIM-BPs were removed showed that there are no significant differences between knockout animals and wild-type controls (Acuna et al., 2015). These studies suggested that synaptic proteins coordinate to regulate the formation and function of synapses.

Through ion channels such as NR2B (*Grin2b*) that are located in the post-synaptic region, Sheng and colleagues performed yeast hybridization and GST-pull-down assays to uncover the interactomes of post-synaptic proteins. Using the cytoplasmic domain of the NR2B subunit of NMDA receptors, Sheng and colleagues discovered that PSD95 is the scaffold protein for NR2B (Cho et al., 1992). PSD95 contains three PDZ (PSD95, Dlg4, and ZO-1) domains, one SH3 domain, and one GK domain (Figure 1), indicating that it organizes the synaptic proteins at the post-synaptic site. Indeed, further studies have demonstrated that it interacts with GKAP through its GK domain (Kim et al., 1997), while GKAP interacts with SHANK proteins in the PDZ domains (Naisbitt et al., 1999). SHANK proteins are also synaptic scaffold proteins that contain PDZ domains and other domains (Figure 1). They interact with Homer (Brakeman et al., 1997), which on the other hand interacts with mGluR and IR3 receptors. The PDZ domains of PSD95 also interact with the protein TARP, which is an auxiliary subunit of AMPAR receptors. In such a way, PSD95 acts as a core that enables the coupling between different kinds of receptors and controls the excitability of synapses. In order to do so, PSD95 clusters together through the N-terminal cysteine formed a di-sulfate bond, which is regulated by the phosphorylation of CDK5 (Morabito et al., 2004). PSD95 may also form clusters with other members in the same family and thus increase the diversity of molecules that can be anchored within synapses (Kim et al., 1996). Additionally, PSD95 interacts with SPAR (Spine-associated RapGAP) via its GK domain (Pak et al., 2001). Overexpression of SPAR in cultured hippocampal neurons increases both the density and diameter of dendritic spines, likely through its effect on the re-organization of the actin cytoskeleton. In contrast, the PDZ domains of PSD95 interact with SynGAP1 which is a RasGAP to restrict spine maturation (Chen et al., 1998; Kim et al., 1998; Clement et al., 2012).

### Assembly of the synaptic proteins during synaptogenesis

#### Trafficking of synaptic proteins

Synaptic proteins need to be transported to the synaptic terminals to execute their synaptic functions (Ahmari et al., 2000; Zhai et al., 2001). In neurons, the anterograde trafficking of cargos through microtubules is mainly achieved through the Kinesin family motor proteins (Hirokawa and Takemura, 2005). Mutations in the kinesin family gene have been shown to cause deficits in synaptic vesicle trafficking (Hall and Hedgecock, 1991; Yonekawa et al., 1998; Pack-Chung et al., 2007). Liprin-α interacts directly with KIF1A through its coiled-coil domain (Shin et al., 2003; Miller et al., 2005), and mutations in Liprin-α showed decreased anterograde movement of synaptic vesicles (Miller et al., 2005). Interestingly, in *unc-104*/KIF1A mutated worms, the localization of syd-2 is unchanged (Zhen and Jin, 1999), suggesting other distinct mechanisms underlying its trafficking. Further studies have shown that to ensure the synaptic localization of *syd-2*, its retrograde trafficking by dynein was inhibited by cyclin-dependent kinases *pct-1* and *cdk-5* (Ou et al., 2010). In addition, the cyclin-dependent kinase 5 pathway promotes synaptogenesis by facilitating the trafficking of synaptic vesicles to the newly formed synapses through KIF1A (Park et al., 2011). Transportation of Piccolo–Bassoon vesicles is mediated by KIF5B which also mediates the activity-dependent pre-synaptic assembly (Cai et al., 2007). The interactions between Bassoon and dynein light chains mediate the retrograde trafficking of vesicles (Fejtova et al., 2009). When fused with fluorescent proteins or through antibody labeling, these synaptic proteins provide important insights into how pre-synaptic and post-synaptic assembly happen, both spatially and temporally. For example, Bassoon clustering occurs in parallel with both inhibitory and excitatory synaptogenesis (Zhai et al., 2000). Piccolo and Bassoon are transported in a protein complex associated with vesicles, which may be the basis for the formation of the active zone (Zhai et al., 2001). In contrast, PSD95 accumulates rapidly after the establishment of new contacts (Bresler et al., 2001), and the post-synaptic scaffold proteins are recruited to the synaptic sites gradually (Bresler et al., 2004).

#### Enrichment of the synaptic proteins by phase separation

These complex synaptic machinery must deposit at specific locations during synaptogenesis. The theory of liquid–liquid phase separation provides insights into how this phenomenon may occur. Recently, it has been shown that both SYD-2 and ELKS can undergo liquid–liquid phase separation before condensation and that such a step is essential for recruiting other active zone proteins (McDonald et al., 2020). The mechanisms underlying the liquid–liquid phase separation seem to be initialized by the oligomerization of Liprin-α followed by the phase separation of ELKS and the recruitment of RIM-α to the condensates (Liang et al., 2021). At the post-synaptic compartment, PSD95 and SynGAP1 interactions have also been reported to be essential for the formation of liquid condensates (Zeng et al., 2016). In addition, both the combinations of PSD95-GKAP and SHANK-Homer are able to form condensed droplets and recruit their interacting proteins, such as NR2B and SynGAP1 (Chen et al., 2020). Given that dendritic spines and axonal buttons are highly specialized compartments, and that the density of these molecules is extremely high, it is reasonable to propose that liquid–liquid phase separation plays an important role in promoting the specification of synapses.

#### Functional studies of the synaptic proteins and their relationships with diseases

**FIGURE 2:**
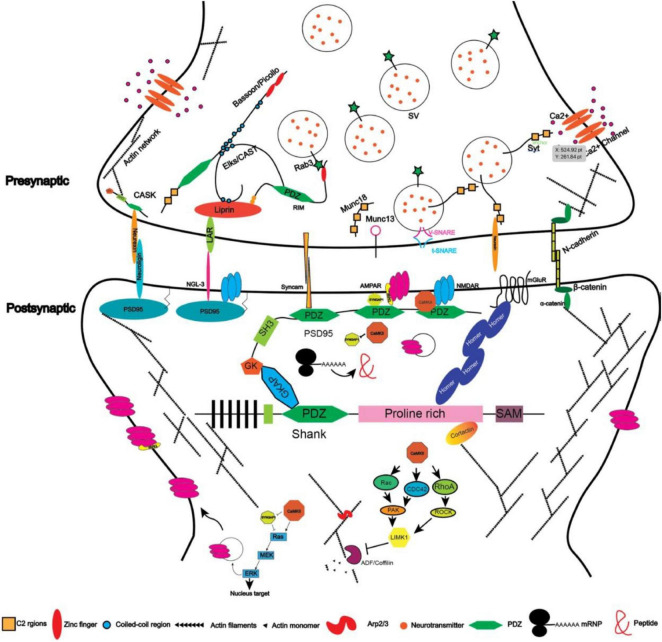
Illustration depicting the protein network in excitatory synapses (protein size not scaled).

Synaptic proteins assemble at synapses through protein–protein interactions and govern synaptic signaling and plasticity (Sheng and Kim, 2002). With accumulated information on these molecules, we are now able to describe a sketch of the synaptic network and “zoom in” to examine their role in synaptogenesis (Figure 2). Since most of the proteins mentioned above are scaffold proteins that provide platforms for the assembly of the synaptic machinery, overexpressing or knocking them down usually affects synaptic strength or morphogenesis (Hung et al., 2008; Wang et al., 2014; Yoon et al., 2021). Mutations in these genes, for example, *SHANK1/2/3* and *SYNGAP1* to name a few, are frequently associated with abnormal synaptogenesis and neurological or psychiatric disorders such as intellectual disability and autism (Table 1).

**TABLE 1:** Scaffold proteins that have been reported to be associated with neurological or psychiatric disorders from human genetics studies.

Genes	Reported disorders and references
*SHANK1*	Autism (Sato et al., 2012)
*SHANK2*	Autism, ID (intellectual disability) (Berkel et al., 2010; Satterstrom et al., 2020)
*SHANK3*	Autism (Durand et al., 2007; Moessner et al., 2007; Satterstrom et al., 2020)
*SYNGAP1*	ID (Intellectual disability) (Hamdan et al., 2009; Rauch et al., 2012; Deciphering Developmental Disorders Study., 2015, 2017), autism (Hamdan et al., 2011; Satterstrom et al., 2020)
*GRIP1*	Autism (Mejias et al., 2011)
*HOMER1*	Autism (Kelleher<suffix>III</suffix>, Geigenmuller et al., 2012), schizophrenia (Norton et al., 2003), depression (Rao et al., 2016)
*PSD95*	Schizophrenia, ID, autism (Coley and Gao, 2018)
*CASK*	ID (Hayashi et al., 2008; Najm et al., 2008), brain malformation (Najm et al., 2008)
*PICCOLO*	Major depressive disorder (Sullivan et al., 2009), bipolar disorder (Choi et al., 2011; Chen et al., 2021), schizophrenia (Chen et al., 2021)
*BASSOON*	Bipolar disorder, schizophrenia (Chen et al., 2021)
*STXBP1/MUNC18-1*	Dravet Syndrome (Carvill et al., 2014), Infantile epileptic encephalopathy (Saitsu et al., 2008)

## Cell adhesion molecules bridging the pre- and post-synaptic machinery during synaptogenesis

Scaffold proteins beneath the synaptic membrane act as the core for the assembly of various molecular modules and thus provide the platform for synaptogenesis. Nevertheless, these platforms must be connected across the synaptic cleft. Cell adhesion molecules are the best candidates to bridge pre- and post-synaptic components. Consistently, it was discovered that cell adhesion molecules are transported together with pre-synaptic components-formed particles, and that cell adhesion molecule also interact with scaffold proteins, suggesting that cell adhesion molecule may mediate the transcellular connections between pre- and post-synaptic terminals (Phillips et al., 2001). Moreover, in artificial synapse-inducing assays, cell adhesion molecules such as *Syncam*1 (Biederer et al., 2002), *Neurexin* (Graf et al., 2004), *Neuroligin* (Scheiffele et al., 2000), and NGL-3 (Woo et al., 2009) have been shown to be sufficient to induce the formation of synapses (Sudhof, 2018), highlighting the importance of cell adhesion molecules. Similarly, evidence from RNAi/shRNA-mediated knockdown and knockout animals also underlines the essential roles of cell adhesion molecules in synaptogenesis. Though these assays have their own caveats, they have provided unique perspectives for our understanding of the processes of synapse formation.

### Leukocyte common antigen-related family receptors protein-tyrosine phosphatases and their ligands/partners

The leukocyte common antigen-related family receptors protein-tyrosine phosphatases (LAR-RPTPs) are composed of extracellular Ig domains and fibronectin domains at the extracellular side, a single transmembrane domain, and a cytoplasmic region that includes two phosphatase domains. The phosphatase domain proximal to the membrane is catalytic active, whereas the distal one is catalytic inactive (Um and Ko, 2013). In vertebrates, there are three genes encoding the proteins of this family: LAR, also called RPTPF; RPTδ, also called PTPRD; and RPTσ, also called RPTPS. They distribute both pre- and post-synaptically. Loss-of-function studies on LAR-RPTPs by RNAi-mediated knocking down resulted in a dramatic reduction in synapse numbers (Dunah et al., 2005). Intracellularly, LAR-RPTPs bind to the scaffold protein Liprin-α and transcellularly, they bind to various cell adhesion molecules including NGL-3, synaptic adhesion-like molecules (SALM), TrkC, and slit and trk like(Slitrks) (Um and Ko, 2013). Their associations with specific cell adhesion molecules are controlled by alternative splicing, indicating functional diversity and specificity (Han et al., 2018). Among them, NGL-3 belongs to the netrin G ligand family and is a single transmembrane protein anchored at the post-synaptic sites through interactions with PSD95. The interaction between LAR and NGL-3 induces the formation of excitatory synapses in cultured neurons (Woo et al., 2009). The phenotypes of mice lacking NGL-3 depends on the genetic background: mice with the C57BL/6J background show reduced growth and abnormal brain anatomy, while mice with a hybrid genetic background exhibit normal gross brain development but impaired synaptic transmission (Lee et al., 2019). SALMs are a family of cell adhesion molecules that also bind to the PDZ domain of PSD95. They have eight leucine-rich repeats, one Ig domain, and one fibronectin domain. Mice lacking SALMs show varying extents of abnormality in synapse development depending on which specific member of the family was knocked out (Lie et al., 2018). SALMs regulate synapse development by inducing the clustering of PSD95 and recruiting NR1 (Lie et al., 2018). Overexpressing and knocking down *Slitrks* can promote and reduce excitatory and inhibitory synapses, respectively, in cultured neurons by interacting with PTPδ and PTPσ (Yim et al., 2013). The detailed interactions among these cell adhesion molecules and their function in synapse development need to be further examined to explain the diverse phenotypes from knockout mice. The complicated interactions will likely compensate for the loss of others. Consistently, mice carrying the mutated *LAR* without the cytoplasmic phosphatase domains exhibited impaired spatial learning and hyperactive behaviors (Kolkman et al., 2004), whereas mice lacking *LAR* showed disrupted glucose homeostasis (Ren et al., 1998). Mice lacking *RPTσ*, on the contrary, showed increased synapse density (Horn et al., 2012). Studies on triple knockout mice either *in vivo* using virus carrying Cre recombinase or in cultured neurons showed that *PTPδ*, *PTPσ*, and *LAR* triple knockout do not affect the development and function of synapses (Sclip and Sudhof, 2020; Emperador-Melero et al., 2021).

### Neurexins and neuroligins

Neurexins were discovered through the chromatography purification of the targets of α-latrotoxin (Ushkaryov et al., 1992). Through the studies of neurexins, Ca^2+^ sensor synaptotagmin was found to interact with their cytoplasmic domains (Brose et al., 1992); the ligands of neurexins, neuroligins, were identified as well (Ichtchenko et al., 1995). In total, there are three neurexin genes, each encoding α- and β- isoforms driven by distinct promoters, and four neuroligin genes with two splice sites differentially distributed in these four genes (Craig and Kang, 2007; Sudhof, 2017). Pre-synaptic neurexins are anchored at the synapses by binding with the scaffold protein CASK, while post-synaptic neuroligins are anchored by binding with PSD95 and gephyrin (Craig and Kang, 2007). Neuroligin 1 is mainly expressed at excitatory synapses, while neuroligin 2 is enriched at inhibitory synapses (Song et al., 1999). When expressed in non-neural cells, neurexins and neuroligins can, respectively, induce the differentiation of post-synaptic and pre-synaptic parts, whereas neuroligins containing autism-associated mutations fail to do so (Scheiffele et al., 2000; Graf et al., 2004; Chubykin et al., 2005; Poulopoulos et al., 2009), suggesting their important roles in the initiation of synaptic terminal specification and synapse formation (Hata et al., 1993a). Consistent with this idea, both loss- and gain-of-function studies have validated the importance of neurexins on synapse formation in cultured neurons (Prange et al., 2004; Chih et al., 2005) and *in vivo* (Chen et al., 2017), though the function of neuroligins on synapse formation *in vivo* was inconsistent with that of *in vitro* studies (Varoqueaux et al., 2006; Shaw and Salmon, 2017). One of the possible reasons for the differential effects of neuroligin 1/2 is that the overexpression effects *in vitro* resulted from the use-dependent stabilization of synapses instead of newly generated ones (Chubykin et al., 2007). These results also suggested that neurexins have other receptors or ligands that are used for synapse formation. Indeed, several lines of evidence demonstrated that neurexins can bind to Cbln1 (Uemura et al., 2010), C1ql2/3 (Matsuda et al., 2016), LRRTM2 (de Wit et al., 2009; Ko et al., 2009; Siddiqui et al., 2010), GABA(A) receptors (Zhang et al., 2010), and latrophilin (Boucard et al., 2012). The interactions between neurexins and these molecules are isoform-specific, and their functions are synaptic type specific (Chih et al., 2006; Sudhof, 2017). For example, neuroligin 1 splice site b determines if it can bind to α-neurexin but does not affect its binding with β-neurexin. In addition, the neuroligin that binds with β-neurexin only promotes synaptogenesis, while the isoform binding to both neurexin isoforms stimulates the enlargement of synapses. LRRTM2 binds to neurexins lacking splice site #4 to induce the formation of excitatory synapses. In line with the diverse interactions of neurexins and neuroligins, various mutations in neuroligins have been reported to be associated with autism disorders; and some syndromic phenotypes can be recapitulated in mice (Sudhof, 2008; Reichelt and Dachtler, 2015).

### N-cadherin

N-cadherins are single transmembrane proteins that localize both pre- and post-synaptically. They consist of extracellular ectodomains that mediate homophilic interactions, a transmembrane domain, and a cytoplasmic domain that connects it to the cytoskeleton system through β- and α-catenins (Bruses, 2006; Figure 2). Evidence supporting N-cadherins’ functions in synaptogenesis include (1) its colocalization with the synaptic marker synaptophysin and PSD95 at the stage of synapse formation (Benson and Tanaka, 1998); (2) its interacting protein β-catenin begins to localize at the dendritic protrusions at the beginning of synaptogenesis in cultured hippocampal neurons, and cultured neurons from β-catenin knockout mice showed thinner dendritic protrusions; and (3) overexpressing the ectodomain deleted N-cadherins in cultured neurons causes the same phenotype as that of the loss of β-catenin in cultured neurons, indicating an essential role of N-cadherins and β-catenin in synapse formation (Togashi et al., 2002; Okuda et al., 2007). However, another study showed that overexpressing only the cytoplasmic domain of N-cadherin in cultured neurons has no effects on either the level of dendritic and synaptic PSD95 and Bassoon but reduced the amplitude of mEPSCs (Peng et al., 2009), suggesting a function of N-cadherin in synaptic transmission. The discrepancies between the two studies might be due to the development stages the experiments were performed with the former at around *div* (days *in vitro*) 21, while the latter at *div*12, suggesting that the functions of N-cadherin vary at different developmental stages. In line with this, knocking down N-cadherin in cultured neurons reduces spine density only at the early stage, suggesting that N-cadherin is required for the initiation but not the maintenance of synapses (Saglietti et al., 2007). Although *in vivo* knockout of N-cadherin in the neocortex and hippocampus disrupted tissue structures such as the cortical lamination and radial glia fiber orientation, there were no obvious effects on spine morphology in cultured hippocampal neurons after Cre mediated knockout in culture (Kadowaki et al., 2007). These results appear to indicate that N-cadherin maybe not essential in the formation of synapses *in vivo* or that there could be other adhesion molecules that can replace N-cadherin to associate with catenins and regulate synapse formation (Yamagata et al., 2018). Nevertheless, it should be noted that acute knockout may take time to remove the protein, and thus when the protein is completely gone, the time window for synaptogenesis may have passed. It is also possible that N-cadherin is important for neural activity-dependent synaptogenesis or pruning. The evidence supporting this hypothesis comes from experiments in which neuronal depolarization, induced by extracellular K^+^, leads to an increased membrane level of N-cadherin. The mechanisms behind this have been reported to be protein kinase D mediated phosphorylation and the ensuing enhanced interaction with β-cateinin (Tanaka et al., 2000; Tan et al., 2010; Bian et al., 2015; Cen et al., 2018). In addition, mutations in β-catenin have been reported to be associated with autism (O’Roak et al., 2012a,b; Sanders et al., 2012; Krumm et al., 2014) and a dominant mutation in humans that causes intellectual disability has been shown to reduce the affinity between β-catenin and N-cadherin (Sanders et al., 2012). These discoveries and the fact that constitutive N-cadherin knockout is embryonic lethal underline the importance of N-cadherin in neural development and suggest that further examination of conditional knockout animals of N-cadherin is necessary for analyzing synaptogenesis.

## The cytoskeleton system as structural support during synaptogenesis

After connections mediated by cell adhesion molecules are established between pre-synaptic terminals and post-synaptic compartments, the highly differentiated sub-cellular regions need to be anchored. The cellular cytoskeleton system provides support to these molecules and thus the building of synapses. We will summarize the function of regulations of cytoskeleton organization.

The actin cytoskeleton is the main cytoskeleton system that supports the structure of the dendritic spines as well as the growth cone of axons (Landis and Reese, 1983). The synaptic scaffold proteins are anchored at the actin framework (Kuriu et al., 2006). However, the actin system is quite dynamic, and the kinetics of actin re-organization underlies the structural changes in dendritic spines (Okabe, 2020). In addition, neural activity also remodels synaptic structure by acting on the actin system (Hotulainen and Hoogenraad, 2010). Actin can be classified as G-actin and F-actin: G-actin is a monomer, while F-actin is a polymer. The balance between G- and F-actin is controlled by actin-associated proteins such as profilin and capping proteins (Amann and Pollard, 2000). The branching of actin filaments is mainly regulated by the Arp2/3 complex while severing filaments and dissociating actin are achieved through the function of cofilin (Amann and Pollard, 2000; Goley and Welch, 2006). The branching dynamics of actin affect the curvature of the plasma membrane, so it determines the fate and shape of the synapses. For example, the head of the dendritic spines usually contains more highly branched actin than the base (Korobova and Svitkina, 2010; Basu and Lamprecht, 2018). Imaging studies also revealed rapid actin dynamics along with the formation of dendritic spines (Fischer et al., 1998). Moreover, when actin undergoes remodeling that is mediated by synaptic activity, it allows the entry of the microtubules into the dendritic spines (Schätzle et al., 2018), possibly facilitating the trafficking of synaptic proteins. Mutations in the actin-regulating molecules impair synaptic plasticity and are associated with neurodevelopmental deficits and cognitive disorders (Bednarek and Caroni, 2011; Lian and Sheen, 2015; Yan et al., 2016; Hlushchenko et al., 2018; Cousin et al., 2021; Qi et al., 2021).

## Molecular mechanisms underlying activity-dependent synaptogenesis

**FIGURE 3:**
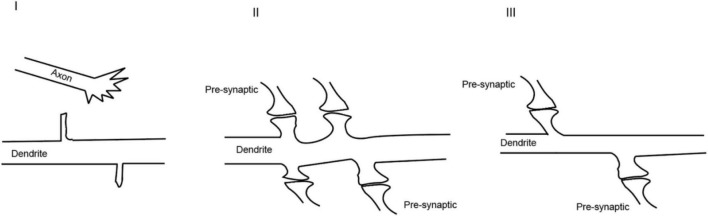
Dynamic process of synaptogenesis: **(I)** axonal and dendritic growth; **(II)** excessive synaptogenesis; **(III)** pruning of the synapses.

In the nervous system, synapses are formed enthusiastically at the beginning which usually culminates with more synapses than required (Figure 3). It is possible that such consequences are supposed to provide enough neural connections for organisms to learn and memorize their encounters. When the organisms gradually adapt to the environments, the synapse network will be reshaped and stabilized by neural activity to operate efficiently. The openings of various channels are the basis for neural activity and will be followed by the spreading of signals from activated synapses to surrounding regions and nuclei. The chemical reactions induced by ion influx or efflux reorganize the structure of synapses (Ebert and Greenberg, 2013).

Studies from the visual system provided a good amount of insights into how neural activities instruct the formation of neural circuits. Reshaping of the neural circuits mediated by neural activity has been demonstrated systematically by studies in the visual system by Hubel and Wiesel (2012) in the 1970s. Parallelly, studies from neuromuscular junctions suggested the essential roles of neural activity in the elimination of muscle innervating axonal terminals of motor neurons (Colman and Lichtman, 1993). Moreover, ocular dominance can form in animals that have undergone binocular deprivation, underscoring the importance of spontaneous activity on synaptogenesis (Shatz, 1990). In a series of pioneering studies led by Katz and Shatz (1996), retinal waves in the early developmental stages were demonstrated to be crucial for the connectivity of retinal ganglion cells to the thalamus. Cholinergic transmission has been shown to be required for the generation of spontaneous waves (Feller et al., 1996). With the advances in imaging techniques and available genetic tools, it has been demonstrated that retinal waves instruct the activity of the midbrain superior colliculus and primary visual cortex (Ackman et al., 2012), and that disrupting the naïve spontaneous activity by deleting the β2-acetylcholine receptor or using optogenetics interferes with the normal retinotopic map refinement (Xu et al., 2011; Zhang et al., 2011; Burbridge et al., 2014).

These studies indicated that the formation of functional synapses occurs at the cost of removing other synapses. However, one of the ensuing questions is whether the reshaping of the neural connectivity happens at the synaptic level and, if yes (Zuo et al., 2005), what are the molecules that are acted on by the neural activity. In addition, evidence on whether there would be *de novo* synaptogenesis after neural activity was still limited at the time. Disrupting neural activity by genetically ablating neurotransmitter release caused a reduction in synapse number, which provided indirect but important evidence supporting the role of neural activity in synaptogenesis (Andreae and Burrone, 2014). Nevertheless, there have been several lines of evidence supporting that there is a cause-and-effect relationship between neural activity and synaptogenesis. First, it has been reported that neural activity can lead to the switch between different types of neurotransmitters (Spitzer, 2017), suggesting that there may be newly generated synaptic types. Second, the treatment of cultured neurons with potassium chloride (KCl) or bicuculline which induces depolarization increased the spine density (Papa and Segal, 1996). This observation was later validated by time-lapse imaging on the same segment of denrites (Hamilton et al., 2012). Third, Maletic-Savatic et al. (1999) observed the rapid formation of dendritic protrusions after tetanic stimulation, certifying that neural activity induces the formation of potential dendritic spines. In addition, Engert and Bonhoeffer performed experiments where they induced neural activity locally and imaged the dynamics of dendritic spines on slices. They found that, accompanied by long-term potentiation induction, there are newly generated spines (Engert and Bonhoeffer, 1999). This study provided evidence that neural activity promotes synaptogenesis within an intact circuit. Recently, it has been corroborated elegantly by the discoveries that the local application of glutamate or GABA with high frequency uncaging at places that originally have no spines will be sufficient to induce the *de novo* formation of dendritic spines in the cortex of developing mice (Kwon and Sabatini, 2011; Oh et al., 2016). Finally, *in vivo* studies suggested strong associations between physiological level neural activity and synaptogenesis. Using two-photon microscopy, it has been shown that there is rapid synapse formation during motor activity in the cortex of developing mice (Xu et al., 2009). At the adult stage, although the turnover speed of dendritic spines becomes lower (Grutzendler et al., 2002), it has also been demonstrated that after stimulation, there exists rapid formation of both excitatory and inhibitory synapses in freely moving animals (Knott et al., 2002). These studies provided compelling evidence that neural activity reshapes the circuits through synaptogenesis. Moreover, the dendritic spines are quite dynamic and can be generated independently or derived from the existing ones by splitting (Zito and Svoboda, 2002). Neural activity can also change the motility of pre-synaptic terminals in a retrograde manner (Gan, 2003; Tashiro et al., 2003). There are a few other models to mimic and study the effects of neural activity on synapse formation, such as environmental enrichment (Kondo et al., 2012) and fear conditioning (Lai et al., 2012) to name a few.

As for the underlying molecular mechanisms for activity-dependent synaptogenesis, multiple aspects are believed to be involved. For example, accumulated evidence shows that neural activity re-organizes the cytoskeleton system and regulates the production of neurotrophic factors from various levels and thus affects synaptogenesis (Ebert and Greenberg, 2013). Most of these mechanistic studies are conducted *in vitro* due to technical obstacles, but there is some *in vivo* evidence as well. We will summarize these investigations here.

### Protein kinases mediated neural activity-dependent synaptogenesis

Under most circumstances, neural activity utilizes Ca^2+^ to activate kinase CaMKII or phosphatases such as calcineurin to affect synaptogenesis (Figure 2). Imaging studies suggested that the activation of CaMKII is restricted to the dendritic spine (Nishiyama and Yasuda, 2015). One of the pathways activated by CaMKII-mediated phosphorylation is the Ras signaling pathway, whose activation results from the inactivation of the RasGAP, Syngap1 (Gamache et al., 2020). On the one hand, the activated Ras can promote the synaptic trafficking of AMPA receptors and enhance transmission efficiency (Zhu et al., 2002). The GluR2 subunit of AMPAR has been reported to interact with the extracellular domain of N-cadherin and promote the morphogenesis of dendritic spines (Passafaro et al., 2003), suggesting function and structure coupling. On the other hand, the activated Ras can relay the signal through PI3K-Akt-mTOR to promote the formation of mushroom-shaped spines (Kumar et al., 2005). The Ras signaling pathway can also send signals to the nucleus by activating ERK, which will phosphorylate CREB (Tang and Yasuda, 2017). Other small GTPases such as Rho/Rac/CDC42 have also been shown to be involved in regulating spine morphology through the cytoskeleton system (Lawler, 1999; Tada and Sheng, 2006; Figure 2). Activated Ras and RhoA signaling can spread to nearby spines whereas the CDC42 signaling is restricted in the activated spines (Nishiyama and Yasuda, 2015). With different temporal kinetics of these three signaling pathways, they coordinated to mediate input-specific structural plasticity of dendritic spines: usually, the signal pathways that will be relayed to nearby synapses or the nucleus have a longer activation time window and vice versa (Hedrick et al., 2016). Of the molecules involved, the loss-of-function mutation of *SYNGAP1* has been reported to be a cause of intellectual disability (Jeyabalan and Clement, 2016). The loss of one copy of *Syngap1* in mice will result in premature development of synapses and impaired synaptic plasticity after stimulation (Clement et al., 2012).

### Transcription and translation for neural activity-dependent synaptogenesis

In 1986, (Greenberg et al., 1986) performed experiments by stimulating the differentiated PC12 cells with a cholinergic agonist and discovered that this treatment induced rapid transcription, which was demonstrated later both in cultured neurons and *in vivo* (Greenberg et al., 1986; Sheng and Greenberg, 1990). These discoveries opened the direction that neural activity might also affect neural connectivity at the level of transcription. Indeed, the signaling from synapses can be transduced to the nucleus to initiate transcription. After the initiation of transcription, these products will be transported to synapses both non-selectively and selectively and act locally at the activated synapses. Multiple mechanisms can contribute to this process such as the cis-elements in the mRNAs (Steward and Worley, 2001), trans-factors such as RNA-binding proteins (Fujii et al., 2005; Doyle and Kiebler, 2011), remodeling of the cytoskeletons system (Schätzle et al., 2018), and local translation and degradation (Tai and Schuman, 2008; Bingol and Sheng, 2011; Glock et al., 2017). In such a way, transcription and local translation supply the materials to support the activity-induced structure changes (Sutton and Schuman, 2006; Ebert and Greenberg, 2013). The effectors that relay information from synapses to the nucleus are usually the calcium-activated CamKII–CamKIV signaling pathways. In this process, a series of transcription factors act coordinately to control the expression of downstream genes to adapt to the external changes as well as to maintain internal homeostasis. Under some circumstances, some membrane-associated proteins will undergo cleavage and translocate into the nucleus to function as transcription factors. For example, the intracellular C-terminal domain of neuregulin 1 has been reported to enhance the transcription of PSD95 in response to elevated neural activity (Bao et al., 2004).

### Neurotrophic factors in neural activity-dependent synaptogenesis

The neural activity also regulates the synapse by regulating neurotrophic factors (Vicario-Abejon et al., 2002). There are four main kinds of neurotrophic factors: NGF, BDNF, NT3, and NT4. The receptors for these neurotrophic factors include TrkA, TrkB, TrkC, and p75NTR. All neurotrophins bind to p75NTR but with lower affinity than to the Trk receptor family. Trk receptors belong to the receptor tyrosine kinase family and, when binding with their ligands these receptors undergo endocytosis and retrogradely relay the neurotrophic factors to the nucleus (Sharma et al., 2010). TrkA is the main receptor for NGF, TrkB for BDNF and NT4, and TrkC for NT3 which can also bind to TrkA and TrkB but with lower efficiency. Neurotrophic factors were recognized as important players in the formation of synapses first at neural muscular junctions and later in the central nervous system as well. Mice with *TrkB* and *TrkC* knockout showed a reduced number of excitatory synapses at the stage of synaptogenesis (Martinez et al., 1998), and mice with conditional deletion of TrkB showed reduced synapse density at the CA1 regions (Luikart et al., 2005). Among the neurotrophic factors, BDNF is the most prominent, and accumulating evidence supports its role in the development of synapses. The mRNA of BDNF can be targeted to dendrites by the alternative 3′UTR (An et al., 2008) and BDNF can mediate neural activity-regulated synapse formation in various ways. First, it can undergo proteolytic maturation after synaptic stimulation (Nagappan et al., 2009; Yang et al., 2009); second, the BDNF receptor TrkB can undergo activity-dependent exocytosis (Lu, 2003); and third, BDNF and its signaling can activate the local translation, thus augmenting the contrast between activated versus non-activated synapses (Kang and Schuman, 1996; Baj et al., 2016). Moreover, the BDNF-TrkB signaling mediated structural change in dendritic spines is NMDAR-CaMKII dependent (Harward et al., 2016).

### Neural activity-induced synaptic scaling through synaptogenesis

Under the influence of neural activity, synaptogenesis not only enables neurons to adapt to new conditions but also acts as a feedback mechanism to maintain neural homeostasis. Activity-dependent neurotransmitter switching is one way to achieve such a goal (Spitzer, 2017). Recently, it has been reported that neural activity can promote the differentiation of parvalbumin interneurons (Donato et al., 2013) and NMDAR-mediated inhibitory potentiation (Chiu et al., 2018). In cultured neurons, elevating neural activity by tetanic stimulation or blocking GABA transmission promotes gephyrin clustering mediated by CaMKII phosphorylation (Flores et al., 2015). At the transcriptional level, transcription factor NPAS4 can maintain the overall activity at a proper level by promoting the transcription of *Bdnf*, thus leading to the formation of inhibitory synapses at somatic regions in excitatory neurons, and excitatory synapses in inhibitory neurons (Lin et al., 2008; Bloodgood et al., 2013; Spiegel et al., 2014). The transcription factor MEF2 can also mediate the activity-induced homeostasis via transcriptional activation of *arc* and *synGAP* to put a restriction on synaptogenesis (Flavell et al., 2006).

## Contributions from glia and microglia during synaptogenesis

Glia is another major cell type in the nervous system other than neurons. Astrocyte is one of the most abundant glia types in the brain. In the central nervous system, the astrocytes are generated after neurogenesis. In studies by Barres and colleagues, it was found that when co-cultured with neurons, astrocytes promoted a higher number of synapses on retinal ganglion neurons (Ullian et al., 2001; Freeman, 2005), and this phenomenon was observed in other types of neurons such as motor neurons (Ullian et al., 2004). Astrocytes regulate synapse formation mainly in a non–cell-autonomous manner, by para-secretion (Christopherson et al., 2005; Eroglu et al., 2009) or through cell adhesion molecules-mediated cell interactions (Stogsdill et al., 2017). In a forward genetic screen in *C. elegans*, it was identified that the glia cells secreted UNC-6 (netrin), which instructed the synaptogenesis between AIY and RIA neurons through regulating pre-synaptic assembly and the axon guidance via its receptor UNC-40 (DCC) (Colon-Ramos et al., 2007). For secreted netrin to act properly, the position of the astrocyte is required to be retained through its interaction with epithelial cells (Shao et al., 2013). The Wnt signaling pathway has also been reported to regulate synaptogenesis via both pre- and post-synaptic sites (Park and Shen, 2012; Shi et al., 2018). These reports highlighted the conserved function of astrocytes on synaptogenesis. In addition, astrocytes can promote microglia-mediated synaptic elimination (Allen and Eroglu, 2017). Though it has been shown that astrocytes are quite heterogeneous across brain regions, if and how astrocytes selectively influence synaptogenesis is still an open question. Moreover, astrocytes can influence synaptic connectivity by modulating the interaction between microglia and neurons. Neurons express the complement system molecules C1q after they were co-cultured with astrocytes, although the mechanisms for astrocytes to stimulate the upregulated C1q remain unknown (Stevens et al., 2007; Stephan et al., 2012). The “tagged” spines will undergo phagocytosis and thus the synaptic elimination by microglia. How the microglia only engulf the synapse and leave the remaining part of the neuron intact is unknown. When this process goes awry, it is usually associated with psychiatric diseases. In a genetic screen for genes associated with the susceptibility to schizophrenia, variants in the complement component *C4* were found to be associated with dysfunction in synapse elimination (Sekar et al., 2016). Overexpression of *C4A/B* causes excess pruning of synapses (Yilmaz et al., 2021) through the phagocytosis function of microglia (Schafer et al., 2012), suggesting their contributions to the pathogenesis of Alzheimer’s disease. Microglia can influence specific types of synapses via both active and feedback mechanisms by responding to changes from the active neurons. For example, it has been shown that neural activity enhances the expression of *Fn14* in thalamic relaying neurons to promote synapse formation; however, at the same time, the expression of *Fn14*’s ligand *TWEAK* increases in microglia, which will restrict the number of spines in relay neurons at places where microglia interact with (Cheadle et al., 2020). It has also been found that microglia can respond to the released GABA neurotransmitter and sculpt the number of synapses (Favuzzi et al., 2021). Neurons express CD47 to exhibit a “don’t eat me” signal and are prevented from being engulfed by microglia (Lehrman et al., 2018). Thus, the homeostatic interactions between astrocytes/microglia and neurons are pivotal for the proper wiring and connectivity of the nervous system (Schafer and Stevens, 2013; Hammond et al., 2018).

## Evolutionary perspectives on molecular mechanisms of synaptogenesis and susceptibility to psychiatric diseases

Given its importance for information processing, the development of the synapse provides an avenue for the understanding of the unrivaled cognitive abilities of humans. It has been observed that accompanied by the increase in the size of the human brain, the spine density is higher in human cortical neurons compared to other primates or rodents (Defelipe, 2011; Schmidt and Polleux, 2021). The most prominent feature of synaptogenesis in humans is the protracted developmental window (Liu et al., 2012). One molecule that has been reported to prevent the premature assembly of a functional synapse is *SYNGAP1*. *SYNGAP1* encodes a RasGAP that negatively regulates the Ras-GTPase activity and thus prevents the formation of actin filaments and the insertion of AMPAR into the synapse. Haploinsufficiency of *SYNGAP1* leads to autism-like spectrum disorders, highlighting the importance of SYNGAP1 dosage in synaptogenesis (Clement et al., 2012). Neurons derived from human iPSCs where the level of SYNGAP1 was reduced showed premature neural activity (LIamosas et al., 2020), further highlighting the temporal importance of synaptogenesis.

Another gene that has been reported to regulate human-specific synaptogenesis is *SRGAP2*. In mice, there is only one copy of *Srgap2*. It contains mainly three functional domains: the F-BAR domains, RhoGAP domain, and SH3 domains. Loss of *Srgap2* in juvenile mice *in vivo* resulted in increased spine density accompanied by decreased spine head width, which commonly reflects the maturation of the synapse. The increase in spine density will persist to adulthood (Charrier et al., 2012), indicating that in mice, Srgap2 restricts the generation but promotes the maturation of synapses. *Srgap2* is required for the maturation of both excitatory and inhibitory synapses at the expense of spine density, and the spine density regulation depends on both the F-bar domain and RhoGAP domain, whereas the regulation of inhibitory synapses density on the dendritic shaft depends on RhoGAP only (Fossati et al., 2016). During evolution, *SRGAP2* genes were duplicated partially three times only in humans which are named *SRGAP2B/C/D*, with *SRGAP2C* expressed at the highest level (Dennis et al., 2012). The product of *SRGAP2C* only contains the F-BAR domain and when overexpressed in mice, it phenocopies the effects of loss of function of *Srgap2A*, suggesting its inhibitory effects on *Srgap2A*. These results suggested that the human-specific duplication generated product prolonged the synaptogenesis of humans. Bioinformatic analysis suggested that chromosome aberrations potentially disrupt the *SRGAP2A* genes that are associated with patients with brain malformations and psychiatric diseases, such as epilepsy, while the diploid copy number of the duplicated gene *SRGAP2C* is highly stable (Dennis et al., 2012).

Recently, it has been reported that in the human prefrontal cortex (PFC), which is an evolutionarily expanded region during evolution and has been reported to have a higher density of synapses, *CBLN2* (cerebellin 2 precursor) is differentially expressed across mice, rhesus macaques, and human (Johnson et al., 2009; Shibata et al., 2021b). Overexpression of CBLN2 in mice is sufficient to increase spinogenesis (Shibata et al., 2021b). *CBLN2* belongs to the cerebellin gene family and is a secreted glycoprotein. It bridges the interaction between neurexins and glutamate receptor σ2, and such a function has been demonstrated in the cerebellum of mice to promote synaptogenesis (Tao et al., 2018). Further examination revealed that the differential expression pattern of *CBLN2* across species is due to the variations in its enhancers that can be regulated by *Sox5* and retinal acid signaling pathways (Tao et al., 2018; Shibata et al., 2021a). Noteworthy, retinal acid signaling pathways have been reported to be associated with psychiatric disorders such as autism spectrum disorders and schizophrenia (Wan et al., 2009; Moreno-Ramos et al., 2015; Huang et al., 2020; Reay and Cairns, 2020). In summary, this evidence not only provides novel insights into the molecular mechanisms of synaptogenesis but also strengthens the associations between abnormal synaptogenesis and psychiatric disorders.

## Concluding remarks and perspectives

Synapse formation is fundamental for the precise wiring of the nervous system. It is a coordinated process that mobilizes various cellular and molecular events. Nevertheless, there are still many unanswered questions in the field. First, it remains unclear how post-synaptic sites are precisely aligned to the pre-synaptic releasing site. Although cell adhesion molecules have provided insightful clues to this question, the *in vivo* functions of these molecules are not always consistent with *in vitro* studies and thus need to be clarified in greater detail. Currently, the widely accepted criteria to define the importance of molecules in synapse development are through genetically modified model organisms or human genetic studies. Second, the temporal sequence of the synapse assembly should be elucidated in more detail. For example, does the synapse formation start from the contact between pre- and post-synaptic sites, or is the cytoskeleton system below the synaptic membrane organized first to prepare a platform for the cell–cell contact? Technical advances for multiplexing labeling and live cell imaging are likely to provide more clues. Third, it is now agreed that synapse formation is not random but rather stereotyped at least in the developmental stage. How the specificity of synaptic contacts is achieved remains largely an open question, especially in mammals. It has been demonstrated in invertebrates that alternative splicing of cell adhesion molecules governs the contact-dependent synaptic specificity, and recently there has been evidence that mice cell adhesion molecules, indeed, govern the wiring specificity in the retina (Sanes and Zipursky, 2020) and hippocampus (Berns et al., 2018; Li et al., 2020). Nevertheless, because of the complexity of the central nervous system, especially the brain, further investigations are still needed to get a comprehensive picture of this landscape. Importantly, it has been shown that most neurological and psychiatric disorders can be attributed to neural developmental disorders, particularly anomalies in synapse development such as synaptic transmission and dendritic spine morphogenesis (Penzes et al., 2011; Zoghbi and Bear, 2012). Studies on how genetic variants contribute to abnormal synapse formation still have a long way to go. With the reduced cost of genomic sequencing and advances in gene therapies, we will be able to unravel the molecular genetics of synapse formation and provide accessible and reliable ways to treat and cure neurological and neuropsychiatric diseases (Sztainberg et al., 2015; Benger et al., 2018; Hill and Meisler, 2021).

## Author contributions

CQ designed the manuscript. CQ and L-DL wrote the manuscript. IF and SM helped to revise the manuscript. All authors contributed to the article and approved the submitted version.
